# Elevated Stearoyl-CoA Desaturase in Brains of Patients with Alzheimer's Disease

**DOI:** 10.1371/journal.pone.0024777

**Published:** 2011-10-26

**Authors:** Giuseppe Astarita, Kwang-Mook Jung, Vitaly Vasilevko, Nicholas V. DiPatrizio, Sarah K. Martin, David H. Cribbs, Elizabeth Head, Carl W. Cotman, Daniele Piomelli

**Affiliations:** 1 Department of Pharmacology, University of California Irvine, Irvine, California, United States of America; 2 Unit of Drug Discovery and Development, Italian Institute of Technology, Genoa, Italy; 3 Institute for Memory Impairments and Neurological Disorders, University of California Irvine, Irvine, California, United States of America; 4 Sanders-Brown Center on Aging, University of Kentucky, Lexington, Kentucky, United States of America; 5 Department of Biological Chemistry, University of California Irvine, Irvine, California, United States of America; Sapienza University of Rome, Italy

## Abstract

The molecular bases of Alzheimer's disease (AD) remain unclear. We used a lipidomic approach to identify lipid abnormalities in the brains of subjects with AD (N = 37) compared to age-matched controls (N = 17). The analyses revealed statistically detectable elevations in levels of non-esterified monounsaturated fatty acids (MUFAs) and mead acid (20:3n-9) in mid-frontal cortex, temporal cortex and hippocampus of AD patients. Further studies showed that brain mRNAs encoding for isoforms of the rate-limiting enzyme in MUFAs biosynthesis, stearoyl-CoA desaturase (SCD-1, SCD-5a and SCD-5b), were elevated in subjects with AD. The monounsaturated/saturated fatty acid ratio (‘desaturation index’) – displayed a strong negative correlation with measures of cognition: the Mini Mental State Examination test (r = −0.80; *P* = 0.0001) and the Boston Naming test (r = −0.57; *P* = 0.0071). Our results reveal a previously unrecognized role for the lipogenic enzyme SCD in AD.

## Introduction

Alzheimer's disease (AD), the most common cause of adult dementia, is characterized by progressive memory impairment, deterioration of language, and visuospatial deficits [1]. Age is the most important factor that predisposes persons to the non-familial (‘sporadic’) form of this disease, which affects an estimated 35 million of elderly people worldwide [2]. It is still unknown how aging interacts with other risk factors of AD – such as abnormal accumulation of Aβ peptides and hyperphosphorylated tau protein in the brain [1]. Nevertheless, it is clear that the development of AD can be influenced by a variety of age-related conditions that are closely associated with systemic dysfunctions in metabolism – including obesity, diabetes and atherosclerosis [3], [4], [5]. In particular, there is evidence suggesting a link between alterations in lipid metabolism and AD. For example, the inheritance of certain isoforms of the lipid-carrier protein, apolipoprotein E (ApoE), is known to increase the risk of AD [6]. Moreover, post mortem analyses of frozen brain samples have documented the existence of multiple lipid abnormalities in AD patients, including changes in ceramides, n-3 polyunsaturated fatty acids (PUFA) and PUFA-derived signaling lipids [7], [8], [9], [10], [11].

In the present study, we utilized a lipidomic approach to identify novel lipid alterations in brain tissue of subjects with AD. We used liquid chromatography/mass spectrometry (LC/MS) to survey frozen brain samples from clinically characterized AD patients and age-matched controls. We found that the levels of nervonic acid (24:1n-9) and other monounsaturated fatty acids (MUFAs) are markedly increased in brain tissue of AD patients. This increase was strongly associated with cognitive dysfunction and was accompanied by enhanced transcription in brain tissue of the rate-limiting enzyme in MUFAs biosynthesis, stearoyl-CoA desaturase (SCD) [12], [13], [14], [15], [16], [17], [18].

## Results

**Figure 1:**
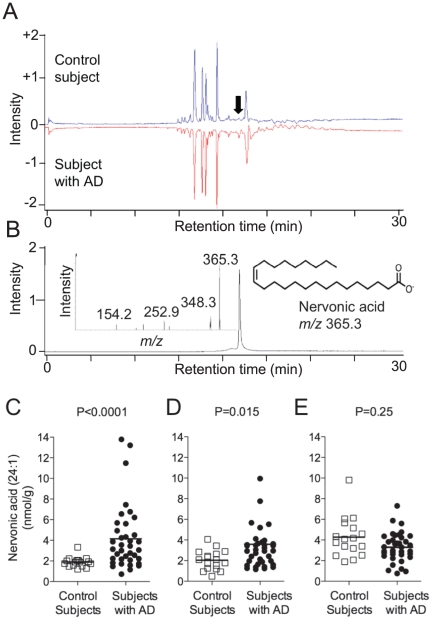
Untargeted lipidomic analyses. Representative mirror-display images and differential LC/MS analysis of lipids from a subject with AD and a control subject. The component detected as being qualitatively different between the two samples (Panel A, arrow), was subsequently identified as nervonic acid, using LC/MS^2^ (Panel B). Quantitative measures of nervonic acid in mid-frontal cortex (Panel C), temporal cortex (Panel D) and cerebellum (Panel E) from control subjects (open squares) and AD patients (closed circles). Two-tailed Welch's *t-*test.

To identify novel molecular alterations in AD, we compared lipid profiles of mid-frontal cortex samples from 17 control subjects and 37 AD patients. The demographic and neuropathological characteristics of the subjects with AD and the control subjects used in this study are shown in Table 1. The groups were closely matched for age and post mortem interval. We extracted tissue lipids with organic solvents and analyzed the extracts by LC/MS using an untargeted approach in which we first acquired data relative to all detectable molecular species of *m/z* 250–500, and then compared data sets obtained from AD and control subjects using algorithms for noise suppression and subtraction of similar molecular features [19]. A representative example of these initial analyses is reported in Fig. 1A. The results suggested that a molecular species of *m/z* 365.3 and retention time 18 min was elevated in AD patients, compared to controls (Fig. 1A, arrow). Further LC/MS^n^ characterization and comparison with an authentic standard identified this molecule as nervonic acid (Fig. 1B), a long-chain MUFA (24:1n-9) that is particularly abundant in brain sphingolipids. Subsequent quantitative measures confirmed that nervonic acid levels were significantly elevated in the mid-frontal and temporal cortices of subjects with AD, relative to control subjects (Fig. 1C,D). No such difference was observed in cerebellum (Fig. 1E).

**Table 1:** Demographic and neuropathological features of the subjects used in this study.

	AD patients	Control subjects
Total number	37 (all Caucasian)	17 (all Caucasian)
Sex (male/female)	20/17	10/7
Age (years)Age range (years)	80.4±7.363–95	80.4±8.564–96
Post mortem interval (hours)	4.2±1.7	4.4±1.5
Plaque score (CERAD)Range: 0–3 (Stage 0-C)	2.76±0.10	0.33±0.21
Tangle score (Braak and Braak)Range: 0–6 (Stage 0-VI)	5.67±0.11	2.33±0.21
MMSE score	11.4±7.2	28.3±1.8
Body weight (lb)	145.3±34.6 (N = 31)	171.2±1.4 (N = 2)
Brain hemisphere (left/right)	19/18	8/7; NA (N = 2)
Freezer storage duration (years)	5.6±2.4	7.6±3.8

Plus-minus values are means ± SD.

Abbreviations: CERAD, Consortium to Establish a Registry for AD; MMSE, Mini-Mental Status Examination; NA, not available.

**Figure 2:**
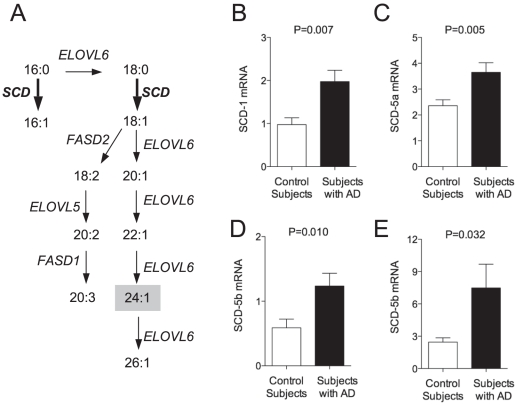
Overview of MUFAs biosynthesis and levels of SCD mRNA in the brain of subjects with AD and control subjects. Palmitic acid (16:0) is transformed into palmitoleic acid (16:1n-7) by the action of SCD (Panel A). Palmitic acid can also be transformed into stearic acid (18:0) by elongase activity (such as those encoded by *ELOVL* genes) and subsequently into oleic acid (18:1) by SCD activity (Panel A). Oleic acid can be then converted into longer chain fatty acids by sequential action of elongases and Δ^5^ and Δ^6^ desaturases (encoded by the *FASD1* and *FASD2* genes, respectively, Panel A). Levels of mRNA for SCD-1 (Panel B), SCD-5a (Panel C) and SCD-5b (Panel D) in hippocampus and SCD-5b (Panel E) in mid-frontal cortex from control subjects (N = 17, open squares) and AD patients (N = 28, closed circles). Two-tailed Welch's *t-*test.

The biosynthesis of nervonic acid starts with the desaturation of stearic acid (18:0) into oleic acid (18:1n-9) catalyzed by SCD activity and proceeds through a series of elongation steps that lead to the production of nervonic and ximenic acid (26:1n-9) (Fig. 2A). Collateral pathways initiated by SCD convert palmitic acid (16:0) into palmitoleic acid (16:1n-7) and stearic acid (18:0) into mead acid (20:3n-9) (Fig. 2A). To determine whether nervonic acid biosynthesis is up-regulated in AD, we quantified these metabolic intermediates and found their levels to be increased in mid-frontal cortex, temporal cortex, and hippocampus of AD patients compared to controls (Tables 2, 3, 4). By contrast, no changes in MUFA content were found in cerebellum (Table 5). Moreover, there were no differences in the levels of saturated fatty acids (SFAs) between subjects with AD and control subjects (Table 2, 3, 4, 5). The ratio between monounsaturated fatty acids (16:1n-7, 18:1n-9) and their saturated precursors (16:0, 18:0) (‘desaturation index’) – was significantly higher in mid-frontal cortex, temporal cortex and hippocampus of subjects with AD compared to control subjects (Table 2, 3, 4). Confirming previous work [20], we recently reported that levels of docosahexaenoic acid (DHA) are decreased in the brains of AD subjects compared to controls [9]. Here, we found a marginal, albeit statistically significant correlation between the desaturation index (16:1/16:0) and DHA levels (P<0.049; r = −0.27) in the mid-frontal cortex of subjects with AD relative to controls.

**Table 2:** Levels of MUFAs and mead acid (nmol/g) in mid-frontal cortex of control subjects and subjects with AD.

Fatty acid	Control subjects	Subjects with AD	Adjusted Difference	P-value
	Mean ± SD; N = 17	Mean ± SD; N = 37	(95% CI)	
16:1	17.51±1.22	21.67±0.89	−4.16 (−7.25, −1.07 )	0.010
18:1	171.70±7.54	193.33±7.5	−21.64 (−43.12, −0.15 )	0.048
20:1	10.11±0.58	12.93±0.95	−2.82 (−5.11, −0.52 )	0.017
22:1	0.85±0.10	1.81±0.25	−0.95 (−1.48, −0.42 )	0.0007
24:1	1.89±0.14	4.16±0.50	−2.26 (−3.29, −1.23 )	<0.0001
26:1	1.50±0.17	3.57±0.40	−2.08 (−2.95, −1.21 )	<0.0001
MUFAs	203.6±9.30	237.5±9.90	−33.91 (−61.30, −6.51 )	0.016
20:3 n-9	4.16±0.33	7.36±0.81	−3.20 (−4.96, −1.43 )	0.0007
16:0	156.20±5.87	162.00±4.98	−5.84 (−21.54, 9.85)	0.45
18:0	252.40±8.67	249.20±8.62	3.22 (−21.54, −27.99)	0.79
20:0	1.55±0.15	1.88±0.11	−0.33 (−0.72, 0.047)	0.083
22:0	0.71±0.10	0.92±0.065	−0.21 (−0.46, −0.043)	0.10
SFAs	409.9±14.90	405.5±14.27	4.39 (−37.56, 46.34)	0.83
16:1/16:0	0.11±0.0040	0.13±0.0040	−0.024 ( −0.037, −0.011 )	0.0008
18:1/18:0	0.68±0.020	0.78±0.022	−0.095 (−0.15, −0.035 )	0.0027
MUFAs/SFAs	0.50±0.015	0.59±0.021	−0.095 (−0.15, −0.042 )	0.0007

Abbreviations: CI, confidence interval. P values for differences between means were computed by linear regression analysis for each fatty acid after adjustment for age, gender, and post mortem interval.

**Table 3:** Levels of MUFAs and mead acid (nmol/g) in temporal cortex of control subjects and subjects with AD.

Fatty acid	Control subjects	Subjects with AD	Adjusted Difference	P-value
	Mean ± SD; N = 17	Mean ± SD; N = 37	(95% CI)	
16:1	14.18±1.07	14.62±0.71	−0.43 (−3.10, 2.23)	0.74
18:1	192.5±10.35	190.2±8.04	2.34 (−24.56, 29.23)	0.86
20:1	9.64±0.81	11.09±1.15	−1.45 (−4.30, 1.40)	0.31
22:1	2.11±0.22	2.51±0.31	−0.40 (−1.17, 0.38)	0.31
24:1	2.00±0.30	3.59±0.55	−1.59 (−2.87, −0.32)	0.015
26:1	1.11±0.30	2.26±0.48	−1.14 (−2.29, 0.0012)	0.050
MUFAs	221.6±12.19	224.7±10.43	−3.09 (−35.85, 29.67)	0.85
20:3 n-9	4.07±0.42	6.24±0.70	−2.18 (−3.81, −0.54)	0.010
16:0	164.2±7.80	153.2±5.32	10.93 (−8.60, 30.46)	0.26
18:0	279.3±16.66	249.2±9.37	30.08 (−9.80, 69.95)	0.13
20:0	2.31±0.24	2.20±0.14	0.12 (−0.46, 0.70)	0.68
22:0	2.03±0.82	1.21±0.17	0.82 (−0.99, 2.64)	0.35
SFAs	447.8±24.15	405.9±14.22	41.94 (−16.53, 100.4)	0.15
16:1/16:0	0.083±0.0040	0.096±0.0033	−0.012 (−0.023, −0.0016)	0.025
18:1/18:0	0.70±0.024	0.78±0.028	−0.079 (−0.15, 0.0058)	0.035
MUFAs/SFAs	0.50±0.014	0.56±0.020	−0.061 (−0.11, −0.013)	0.014

Abbreviations: CI, confidence interval. P values for differences between means were computed by linear regression analysis for each fatty acid after adjustment for age, gender, and post mortem interval.

**Table 4:** Levels of MUFAs and mead acid (nmol/g) in hippocampus of control subjects and subjects with AD.

Fatty acid	Control subjects	Subjects with AD	Adjusted Difference	P-value
	Mean ± SD; N = 17	Mean ± SD; N = 37	(95% CI)	
16:1	8.70±0.68	19.80±1.04	−11.10 (−13.61, −8.58 )	<0.0001
18:1	141.3±13.27	180.7±10.95	−39.39 (−74.29, −4.48)	0.028
20:1	5.82±0.43	8.21±065	−2.40 (−3.99, −0.80)	0.0042
22:1	2.27±0.33	2.75±0.36	−0.47 (−1.46, 0.51)	0.34
24:1	4.27±0.93	3.40±0.44	0.86 (−1.26, 2.99)	0.41
26:1	6.35±1.34	4.82±0.72	1.54 (−1.67, 4.74)	0.33
MUFAs	167.7±15.73	219.6±13.35	−51.99 (−93.85, −10.12)	0.029
20:3 n-9	15.34±2.11	22.98±2.41	−7.63 (−14.10, 1.17)	0.022
16:0	154.80±10.50	171.50±7.44	−16.67 (−42.67, 9.57)	0.20
18:0	262.80±21.19	241.31±11.16	21.53 (−28.80, 70.85)	0.38
20:0	5.78±0.68	4.03±0.21	1.75 (−0.28, 3.23)	0.022
22:0	1.99±0.33	1.19±0.074	0.80 ( 0.092, 1.50)	0.029
SFAs	425.4±32.10	418.01±18.46	7.41 (−68.72, 83.55)	0.84
16:1/16:0	0.058±0.0044	0.12±0.0049	−0.059 (−0.072, −0.046)	<0.0001
18:1/18:0	0.55±0.047	0.75±0.030	−0.20 (−0.31, −0.085)	0.0013
MUFAs/SFAs	0.41±0.0038	0.52±0.0020	−0.11 (−0.20, −0.032)	0.0086

Abbreviations: CI, confidence interval. P-values for differences between means were computed by linear regression analysis for each fatty acid after adjustment for age, gender, and post mortem interval.

**Table 5:** Levels of MUFAs and mead acid (nmol/g) in cerebellum of control subjects and subjects with AD.

Fatty acid	Control subjects	Subjects with AD	Adjusted Difference	P-value
	Mean ± SD; N = 17	Mean ± SD; N = 37	(95% CI)	
16:1	16.45±1.34	17.42±0.60	−0.97 (−4.012, 2.07)	0.52
18:1	253.2±18.90	230.3±6.84	22.83 (−19.25, 64.90)	0.27
20:1	17.33±1.82	15.47±0.78	−2.26 (−2.26, 5.98)	0.36
22:1	2.71±0.29	3.40±0.65	−0.68 (−2.12, 0.76)	0.34
24:1	4.3±0.50	3.56±0.34	0.71 (−0.52, 1.94)	0.25
26:1	4.16±0.55	4.32±0.57	−0.15 (−1.757, 1.45)	0.85
MUFAs	290.2±22.27	273.7±8.09	16.44 (−32.99, 65.88)	0.50
20:3 n-9	6.16±0.65	7.60±0.43	−1.43 (−3.03, 0.16)	0.077
16:0	204.1±18.36	198.3±7.87	5.85 (−35.95, 47.65)	0.77
18:0	395.7±26.25	367.4±11.90	28.35 (−31.78, 88.48)	0.34
20:0	3.89±0.44	3.33±0.29	0.56 (−0.53, 1.64)	0.30
22:0	1.71±0.21	1.57±0.16	0.14 (−0.42, 0.69)	0.62
SFAs	605.5±43.45	570.6±17.63	34.89 (−63.62, 133.4)	0.47
16:1/16:0	0.080±0.0047	0.092±0.0025	−0.011 (−0.022, −0.00022)	0.046
18:1/18:0	0.62±0.029	0.63±0.014	−0.010 (−0.077, 0.057)	0.76
MUFAs/SFAs	0.48±0.021	0.48±0.0092	−0.0019 (−0.049, 0.046)	0.94

Abbreviations: CI, confidence interval. P values for differences between means were computed by linear regression analysis for each fatty acid after adjustment for age, gender, and post mortem interval.

These results identify a previously unrecognized increase in MUFAs levels in the brains of AD patients, suggesting that brain SCD expression might also be elevated in these subjects. This possibility was confirmed by quantitative RT-PCR analyses, which showed that levels of mRNA encoding for SCD-1, SCD-5a and SCD-5b – three SCD isoforms present in the human brain [13], [21] – were elevated in hippocampus of AD patients (Fig. 2B–D). In mid-frontal cortex, only the mRNA encoding for SCD-5b was found to be increased (Fig. 2E).

**Figure 3:**
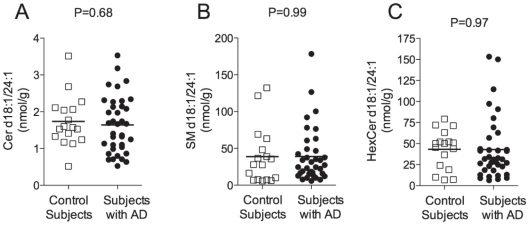
Levels of nervonic acid-containing lipids in the brain of subjects with AD and control subjects. Levels of ceramide d18:1/24:1 (Panel A), sphingomyelin d18:1/24:1 (Panel B), hexosylceramide d18:1/24:1 (Panel C) in mid-frontal cortex from control subjects (N = 17, open squares) and AD patients (N = 37, closed circles). Two-tailed Welch's *t-*test.

To determine whether the increase in free nervonic acid is due to an up-regulation of MUFA biosynthesis or MUFA release from sphingolipids, we measured the levels of nervonic acid-containing molecular species in the mid-frontal cortex of subjects with AD compared to control subjects. No changes were observed in the levels of selected ceramide, sphingomyelin, and cerebroside species (Fig. 3). These findings support the conclusion that AD might be associated with an up-regulation of MUFA biosynthesis, rather than a non-specific alteration in sphingolipid content. We cannot exclude, however, the possibility that the effects observed here might be due, at least in part, to changes in the gray/white matter ratio between controls and subjects with AD.

**Figure 4:**
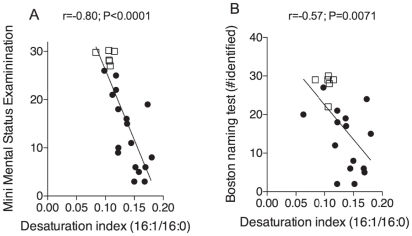
Correlation between brain MUFA levels and cognitive function in subjects with AD and control subjects. Correlation between desaturation index (16:1/16:0) in mid-frontal cortex and most recent neuropsychometric scores from control subjects (open squares) and subjects with AD (closed circles). There were significant correlations between individual desaturation indices and available Mini-Mental State Examination scores (Panel A) and Boston Naming Test scores (Panel B). Partial correlation analysis after adjustment for age, gender and post mortem interval.

Next, to explore the clinical significance of our findings, we searched for statistical correlations between MUFA levels and measures of cognitive function. We found that the desaturation index (16:1/16:0) in mid-frontal cortex displayed a strong negative correlation with cognitive scores from two standard neuropsychometric tests: the Mini-Mental Status Examination test (which assesses global cognition; r = −0.80; P = 0.0001) and the Boston Naming test (which assesses language facility; r = −0.57; P = 0.0071) (Fig. 4). These findings point to the existence of a functional link between heightened MUFA levels in the brain and deterioration of mental functions in subjects with AD. The results do not establish, however, whether this link represents causation, and do not elucidate the molecular mechanisms responsible for increased MUFA levels in AD.

**Figure 5:**
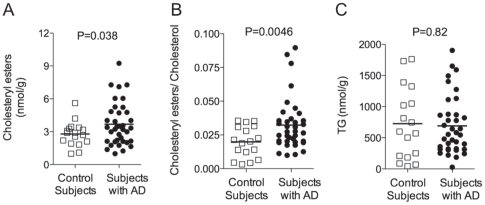
Levels of cholesteryl esters and TG in the brain of subjects with AD and control subjects. Levels of total cholesteryl esters (Panel A), cholesteryl esters to cholesterol ratio (Panel B) and total TGs (Panel C) in mid-frontal cortex from control subjects (N = 17, open squares) and AD patients (N = 37, closed circles). Two-tailed Welch's *t-*test.

SCD is a key lipogenic enzyme. Indeed, by converting SFAs into MUFAs, SCD provides preferred substrates for the acyl-CoA:cholesterol acyl transferase (ACAT), an enzyme that catalyzes the conversion of cholesterol into cholesteryl esters. ACAT inhibitors are potent hypolipidemic and antiatherosclerotic drugs, which might also reduce AD pathology [22], [23], [24]. To determine whether the observed increase in MUFAs availability affected cholesterol homeostasis in AD, we measured the levels of both cholesteryl esters and cholesterol by LC/MS. We found that the levels of cholesteryl esters and the cholesteryl esters/cholesterol ratio were increased in mid-frontal cortex of subjects with AD compared to control subjects (Fig. 5). These results highlight a potential role played by SCD in the regulation of cholesterol homeostasis in AD brain. In contrast, no changes were observed for total triglycerides (TG) in mid-frontal cortex of subjects with AD compared to control subjects [25], [26].

## Discussion

Our results identify a previously unrecognized alteration in the neural lipidome, which appears to be associated with AD. Using an untargeted lipidomic strategy, we found that the levels of nervonic acid and other MUFAs produced by SCD are markedly elevated in frozen brain samples from AD patients, compared to age-matched control subjects, and that these changes strongly correlate with cognitive impairment. We further found that the transcription of three SCD isoforms present in the human brain – SCD-1, SCD-5a and SCD-5b – is heightened in AD. These findings reveal an unexpected role for brain MUFAs and SCD in AD.

Although the functional consequences of elevated brain MUFA levels are unclear at present, peripheral SCD is recognized to be a critical control point in the development of metabolic diseases. Elevated levels of SCD activity and desaturation index appear to be associated with key aspects of the metabolic syndrome – insulin resistance, abdominal adiposity and hyperlipidemia – and have been proposed as biological markers for this condition [13], [14], [15], [17], [18], [26]. Furthermore, animal studies have shown that SCD-derived palmitoleate (16:1n-7) participates as a blood-borne messenger in the control of energy homeostasis and insulin resistance [27]. Because of these discoveries, SCD has recently emerged as a possible target for the treatment of diabetes, hyperlipidemia and obesity [28], [29], [30].

Two distinct SCD isoforms (SCD-1 and SCD-5) are expressed in human tissues, but little is known about the substrate specificity and functional roles of these variants. Human SCD-1 shares about 85% amino-acid identity with the four known rodent isoforms and is highly expressed in brain, liver and adipose tissue [31]. By contrast, human SCD-5 isoforms have limited homology with rodent SCD and are predominantly expressed in brain and pancreas [21], [32]. Previously studies have documented a progressive increase in MUFA levels and SCD-1 expression in human frontal cortex during normal aging and in patients with bipolar disorder [33]. Such an increase was paralleled by lowered levels of the n-3 polyunsaturated fatty acid, DHA [34], which is consistent with the ability of polyunsaturated fatty acids (PUFAs) to negatively regulate SCD expression [35]. In agreement with those data, previous work from our laboratory and others has reported that free DHA levels are markedly decreased in the brain of subjects with AD [9], [20].

Notably, the up-regulation of MUFA levels was accompanied by an increase in mead acid (20:3n-9) (Tables 2, 3, 4), which is the only non-essential PUFA that can be synthesized through a de novo pathway, in which SCD appear to be the rate-limiting enzyme (Fig. 2A). In conditions of DHA deficit, mead acid could contribute to maintain a homeostasis in the unsaturation level of the cellular membranes. Therefore, together with increased MUFA levels and decreased DHA levels, an elevation in the levels of mead acid might serve as an additional biological marker for AD.

As with all observational studies, there is the possibility that unmeasured factors may explain our results, even though we chose a priori to adjust for the three most likely confounders – age, gender and post mortem interval. For example, medication history was only partly available for our subject groups and additional work is needed, therefore, to evaluate its potential impact. Furthermore, although we paid particular attention in using exclusively gray matter that was appropriately dissected by an experienced neuropathologist, we cannot exclude the possibility that altered axonal myelination could contribute to the elevation in MUFA levels observed in AD brains. Despite these limitations, our study does point to a previously unrecognized connection between cognitive impairment in AD and up-regulation of the lipogenic enzyme SCD. Future investigations need to explore the molecular basis for this putative association as well as its possible implications in the diagnosis and treatment of AD.

## Materials and Methods

### Human brain tissue procurement

We used frozen brain samples from a total of 17 non-demented control subjects and 37 pathologically confirmed subjects with AD (males/females: control subjects, 10/7; subjects with AD, 20/17), provided by the Institute for Brain Aging and the Dementia and Alzheimer's Disease Research Center at the University of California, Irvine. Average sample weight was 36.8±1.0 mg, mean±SEM (N = 54). Four brain areas rich in gray matter were selected for analysis: mid-frontal cortex (Brodmann area 9), temporal cortex (Brodmann area 20), hippocampus (Brodmann area 34) and cerebellum. Subjects were matched for age (in years: control subjects, 80.4±8.5; subjects with AD, 80.4±7.3) and post mortem interval (in hours: control subjects, 4.4±1.5; subjects with AD, 4.2±1.7). AD cases met the National Institute on Aging-Reagan Institute criteria for intermediate or high likelihood of AD. Psychometric tests were used for correlative analyses when available and were assessed 44.3±35.9 months before death for control subjects and 11.0±6.2 months before death for subjects with AD. All subjects and their caregivers (when appropriate) provided informed written consent for both the clinical examination as well as for brain donation at the University of California Irvine. The protocols and informed consent have been approved by the University of California Irvine Institutional Biosafety Committee.

### Lipid extractions

Lipid extractions were conducted as described [36]. Briefly, frozen brain samples were rapidly weighed and homogenized in ice-cold methanol containing appropriate internal standards (listed below). Lipids were extracted by adding chloroform and water (2/1, vol/vol) and fractionated through open-bed silica gel columns by progressive elution with chloroform/methanol mixtures. Fractions eluted from the columns were dried under nitrogen, reconstituted in chloroform/methanol (1∶4, vol/vol; 0.1 ml) and subjected to LC/MS.

### Untargeted lipidomic analyses

Unbiased lipidome-wide analyses were conducted using an Agilent 1100-LC system coupled to an ion-trap XCT mass detector equipped with an electrospray ionization (ESI) interface (Agilent Technologies, Inc., Palo Alto, CA). Lipid extracts were separated on a XDB Eclipse C18 column (50×4.6 mm i.d., 1.8 µm, Zorbax, Agilent Technologies) and they were eluted with a gradient of methanol in water (from 60% to 100% methanol in 20 min) at a flow rate of 1 ml/min. Column temperature was kept at 30°C. Capillary voltage was set at 3 kV and fragmentor voltage was 120 V. N_2_ was used as drying gas at a flow rate of 13 liters/min and a temperature of 350°C. Nebulizer pressure was set at 60 PSI. Lipids were analyzed in full scan mode from 250 to 500 mass-to-charge ratio (*m/z*) in both positive and negative ionization mode. Data analyses, which consisted in application of algorithms for noise and background reduction and differential comparative analyses, were performed using MS Processor from Advanced Chemistry Development, Inc. (Toronto, Canada). The COMPARELCMS input parameters were as follows: MCQ threshold = 0; Smoothing Window Width = 3; Noise Level = 20 scans; Peak Width = 20 scans; Delta Scan Level = 3 scans; Ratio Level = 5; Additional Data Reduction = ON; Baseline Correction = OFF. The processing took approximately 120 seconds on a Pentium IV 1.5 GHz computer.

### Targeted lipidomic analyses

Lipid molecular species were quantified by normalizing the individual molecular ion peak intensity with an internal standard for each lipid class. A mixture of non-endogenous molecules was used as internal standards and added before the extraction process to allow lipid levels to be normalized for both extraction efficiency and instrument response.

#### Fatty acids

Fatty acids were quantified with an Agilent 1100 liquid chromatograph coupled to a 1946D mass detector equipped with an electrospray ionization interface (Agilent Technologies, Palo Alto, CA). A reversed-phase XDB Eclipse C18 column (50×4.6 mm i.d., 1.8 µm, Zorbax, Agilent Technologies) was eluted with a linear gradient from 90% to 100% of A in B for 2.5 min at a flow rate of 1.5 ml/min with column temperature at 40°C. Mobile phase A consisted of methanol containing 0.25% acetic acid and 5 mM ammonium acetate; mobile phase B consisted of water containing 0.25% acetic acid and 5 mM ammonium acetate. Column temperature was kept at 40°C. Mass detection was in the negative ionization mode, capillary voltage was set at −4.0 kV and fragmentor voltage was 120 V. Nitrogen was used as drying gas at a flow rate of 13 liters/min and a temperature of 350°C. Nebulizer pressure was set at 60 pounds per square inch. For quantification purposes, the deprotonated pseudo-molecular ions [M-H]^−^ of the fatty acids were monitored in the selected ion-monitoring mode, using d_8_-arachidonic acid (Cayman Chemical, Ann Arbor, MI) as internal standard (*m/z* = 311.3). Commercially available fatty acids (Nu-Chek Prep, Elysian, MN, Cayman Chemical or Sigma-Aldrich, St Louis, MO) were used as references.

#### Sphingolipids

Sphingolipid molecular species were analyzed by tandem mass spectrometry, using an Agilent 1100 liquid chromatograph coupled to an electrospray ionization-ion-trap XCT mass detector. Ceramides were separated on a Poroshell 300 SB C18 column (2.1×75 mm i.d., 5 µm; Agilent Technologies) maintained at 30°C. A linear gradient of methanol in water containing 5 mM ammonium acetate and 0.25% acetic acid (from 80% to 100% of methanol in 3 min) was applied at a flow rate of 1 ml/min. Detection was in the positive mode, capillary voltage was 4.5 kV, skim1 −40 V, and capillary exit −151 V. N_2_ was used as drying gas at a flow rate of 12 L/min, temperature of 350°C, and nebulizer pressure of 80 psi. Helium was used as collision gas. Nervonic-acid containing ceramide was identified by comparison of its LC retention time and MS^n^ fragmentation pattern with that of an authentic standard (Avanti Polar Lipids). Extracted ion chromatograms were used to quantify ceramide d18:1/24:1 [M+H]^+^ (*m/z* = 648.6>630.8>264.3), using ceramide d18:1/12:0 [M+H]^+^ (*m/z* = 482.5>464.5>264.3) as an internal standard. Complex sphingolipids were separated using a reversed-phase Poroshell 300SB C18 column (2.1×75 mm i.d., 5 µm, Agilent) and eluted with a linear gradient from 85% to 100% of mobile phase A in B in 5 min at a flow rate of 1.0 ml/min with column temperature at 50°C. Mobile phase composition was as described above. The capillary voltage was set at −4.0 kV and skimmer voltage at −40 V. Nitrogen was utilized as drying gas at a flow rate of 10 liters/min, temperature at 350°C and nebulizer pressure at 60 pounds per square inch. Helium was the collision gas and fragmentation amplitude was set at 1.2 V. Mass detection was set in either positive or negative ionization mode and was controlled by the Agilent/Bruker Daltonics software version 5.2. Sphingolipids were identified using reference standards (Avanti Polar Lipids) and quantified by multiple reaction monitoring as follows: sphingomyelin d18:1/24:1 [M]^+^ (*m/z* = 813.8>754.8), hexosylceramide d18:1/24:1 [M-H]^−^ (*m/z* = 808.8), using sphingomyelin d18:1/12:0 [M]^+^ (*m/z* = 647.8>588.8) and glucosylceramide d18:1/12:0 [M-H]^−^ (*m/z* = 642.8) as internal standards.

#### Cholesterol and triacylglycerols (TGs)

We used an Agilent 1100-LC system coupled to a MS detector Ion-Trap XCT interfaced with atmospheric pressure chemical ionization (Agilent Technologies). Lipids were separated on a Poroshell 300SB C18 column (2.1×75 mm i.d., 5 µm, Agilent Technologies) at 50°C. A linear gradient of methanol in water containing 5 mM ammonium acetate and 0.25% acetic acid (from 85% to 100% of methanol in 4 min) was applied at a flow rate of 1 ml/min. MS detection was set in positive mode. Corona discharge needle voltage set at 4 kV. Capillary voltage was 4.0 kV, skim1 40 V, and capillary exit at 118 V. Nitrogen was used as drying gas at a flow rate of 10 liters/min, temperature of 350°C, nebulizer pressure of 50 PSI and vaporization temperature at 400°C. Helium was used as collision gas. Cholesterol and total cholesteryl esters were detected at *m/z* 369.3 ([cholesterol+H-H_2_O]^+^). Total TGs were quantified by integrating the area of the total ion current (*m/z* 700–900) at a selected interval of retention time (from 4 to 5 min), using TG 19:1/19:1/19:1 (*m/z* 944.8, Nu-Chek Prep) as an internal standard.

### Gene expression

Total RNA was extracted from 10–50 mg of frozen brain tissue using TRIzol reagent (Invitrogen, Carlsbad, CA) and was purified with the RNeasy mini kit (Qiagen, Valencia, CA). RNA quality was assessed using an Agilent BioAnalyzer and by UV spectrophotometry. First-strand complementary DNAs were synthesized using SuperScript II RNaseH reverse transcriptase (Invitrogen). Reverse transcription of total RNA (2 µg) was carried out using oligo(dT)12–18 primers for 50 min at 42°C. mRNA levels were measured by quantitative real-time polymerase chain reaction (RT-PCR) with a Mx 3000P system (Stratagene, La Jolla, CA). The following primers and fluorogenic probes were purchased from Applied Biosystems (TaqMan Gene Expression Assays, Foster City, CA): *SCD-1*, Hs01682761_m1; *SCD-5 transcript variant 1* (*SCD-5a*, NCBI Refseq#NM_001037582), Hs01125695_m1; *SCD-5 transcript variant 2* (*SCD-5b*, NCBI Refseq#NM_024906), Hs01125106_m1. RNA levels were normalized using glyceraldehyde 3-phosphate dehydrogenase (*GAPDH*) as standard.

### Statistical analyses

Descriptive statistics are presented as means ± SD. The differences between unadjusted mean values were determined by two-tailed Welch's *t-*test. Associations between parameters were tested by partial correlation analysis (Pearson's). We used linear regression to estimate the association between individual lipid species and AD, adjusting for age, gender, and post mortem interval. All confidence intervals correspond to a 95% confidence level.
